# Identifying candidates for targeted gait rehabilitation after stroke: better prediction through biomechanics-informed characterization

**DOI:** 10.1186/s12984-016-0188-8

**Published:** 2016-09-23

**Authors:** Louis N. Awad, Darcy S. Reisman, Ryan T. Pohlig, Stuart A. Binder-Macleod

**Affiliations:** 1 Department of Physical Therapy and Athletic Training, College of Health and Rehabilitation Sciences: Sargent College, Boston University, Boston, MA 02215 USA; 2 Wyss Institute For Biologically Inspired Engineering, Harvard University, Cambridge, MA 02138 USA; 3 Department of Physical Therapy, University of Delaware, Newark, DE 19713 USA; 4 Graduate Program in Biomechanics and Movement Science, University of Delaware, Newark, DE 19713 USA; 5 Delaware Clinical and Translational Research ACCEL Program, Newark, DE 19713 USA; 6 Biostatistics Core Facility, University of Delaware, Newark, DE 19713 USA

**Keywords:** Rehabilitation, Physical Therapy, Walking, Locomotion, Gait, Stroke, Biomechanics, Prediction, Prognostic, Efficacy, FES, Electrical stimulation

## Abstract

**Background:**

Walking speed has been used to predict the efficacy of gait training; however, poststroke motor impairments are heterogeneous and different biomechanical strategies may underlie the same walking speed. Identifying which individuals will respond best to a particular gait rehabilitation program using walking speed alone may thus be limited. The objective of this study was to determine if, beyond walking speed, participants’ baseline ability to generate propulsive force from their paretic limbs (paretic propulsion) influences the improvements in walking speed resulting from a paretic propulsion-targeting gait intervention.

**Methods:**

Twenty seven participants >6 months poststroke underwent a 12-week locomotor training program designed to target deficits in paretic propulsion through the combination of fast walking with functional electrical stimulation to the paretic ankle musculature (FastFES). The relationship between participants’ baseline usual walking speed (UWS_baseline_), maximum walking speed (MWS_baseline_), and paretic propulsion (prop_baseline_) versus improvements in usual walking speed (∆UWS) and maximum walking speed (∆MWS) were evaluated in moderated regression models.

**Results:**

UWS_baseline_ and MWS_baseline_ were, respectively, poor predictors of ΔUWS (*R*^*2*^ = 0.24) and ΔMWS (*R*^*2*^ = 0.01). Paretic propulsion × walking speed interactions (UWS_baseline_ × prop_baseline_ and MWS_baseline_ × prop_baseline_) were observed in each regression model (*R*^*2*^*s* = 0.61 and 0.49 for ∆UWS and ∆MWS, respectively), revealing that slower individuals with higher utilization of the paretic limb for forward propulsion responded best to FastFES training and were the most likely to achieve clinically important differences.

**Conclusions:**

Characterizing participants based on both their walking speed and ability to generate paretic propulsion is a markedly better approach to predicting walking recovery following targeted gait rehabilitation than using walking speed alone.

## Background

Stroke remains a leading cause of long-term disability [1], with the restoration of walking ability being the most commonly voiced goal by stroke survivors [2]. Many factors contribute to the limitations of current interventions [3]. One major factor is the heterogeneity of poststroke motor impairments [4]. Indeed, the effectiveness of targeted interventions may vary across individuals as a function of their baseline abilities [5, 6]. Intervention studies that report outcomes across participants with a wide range of abilities and impairments may not accurately estimate the effects of an intervention for a particular participant. A better understanding of how participants’ baseline walking abilities influence the effects of locomotor intervention would facilitate optimal intervention design and advance individualized, evidence-based rehabilitation efforts in this complex population.

Previous investigators have attempted to address this problem by reporting results across subgroups of participants, with walking speed serving as a common stratification criteria [5, 7–10]. Indeed, walking speed has been named the 6th vital sign [11] for its robust measurement and prediction of walking performance [12], community walking capacity [13], rehabilitation potential [14], and quality of life [15]. However, different motor impairments may result in the same walking speed [16, 17] and changes in walking speed may occur via a variety of biomechanical mechanisms, including the restoration of symmetrical gait mechanics or improved compensatory strategies [16, 18–20]. As such, it is not clear if baseline walking speed alone is a sufficient predictor of the improvements in walking speed that may result from gait interventions, especially those designed to target specific gait deficits. We posit that knowledge of the gait mechanics underlying participants’ walking speed would enhance the ability to identify appropriate candidates for targeted locomotor training.

The most likely factors influencing an intervention’s effects may depend on the particular nature of the intervention. For example, baseline self-efficacy may be the primary moderator of posttraining outcomes for an intervention designed to improve walking ability by improving participants’ balance self-efficacy. The present investigation studies, as a model, a gait intervention designed to target deficits in paretic propulsion through the combination of fast locomotor training with functional electrical stimulation to the paretic ankle musculature during walking (FastFES) [21, 22]. This novel combination therapy is designed to improve an individual’s ability to generate propulsion by synergistically facilitating a more posterior positioning of the paretic trailing limb relative to the body’s center of mass and increased activation of the paretic plantarflexors during late stance—key variables underlying the generation of forward propulsion [23]. A recent randomized controlled trial conducted by our laboratory demonstrated the unique ability of this hypothesis-driven combination therapy to concurrently increase participants’ ability to walk farther distances and walk with a substantially lower energy cost of walking [22]. Based on the FastFES framework [21], we hypothesized that beyond participants’ baseline walking speed, knowledge of participants’ ability to generate paretic propulsion would enhance our ability to identify the best candidates for FastFES training.

## Methods

This is a secondary analysis of data reported in two previous articles studying the FastFES locomotor training program [21, 22]. These articles, as well as prior work from our laboratory [24], can be referenced for greater detail on the participants studied, the FastFES training intervention, and our testing methodology. The 27 individuals included in this report were all those that underwent FastFES locomotor training. Participants were at least 6 months post a single cortical or subcortical stroke, had observable gait deficits but were able to walk for 6 min without the assistance of another individual or orthotic support, had sufficient passive ankle dorsiflexion range of motion to dorsiflex the ankle to neutral with the knee extended, had at least 10° of passive hip extension range of motion, and were able to communicate with investigators and follow instructions. Cerebellar stroke, any condition other than stroke that limited walking ability, neglect or hemianopia, or unexplained dizziness during the prior 6 months each served as exclusion criteria. The University of Delaware’s institutional review board approved the protocol executed for this study. Medical clearance and a submaximal stress test were required prior to the start of training.

### Testing

Clinical and motion analysis evaluations were conducted pretraining, posttraining, and at a 3-month follow-up. The 10-m walk test [25] measured participants’ self-selected, usual (UWS) and maximum (MWS) overground walking speeds, which served as dependent variables. Participants were also tested while walking on an instrumented split-belt treadmill set to speeds similar to their overground UWS. The peak anterior ground reaction force produced by the paretic limb during double support was normalized by bodyweight (%bw) and served to measure participants’ ability to generate forward propulsion during walking (prop_baseline_).

### Analyses

All analyses were performed using SPSS version 23. Paired t-tests tested for pretraining to posttraining and 3-mo follow-up changes in usual and maximum walking speeds. The relationships among participants’ baseline UWS, MWS, prop_baseline_, posttraining changes in UWS (ΔUWS), and posttraining changes in MWS (ΔMWS) were assessed using Pearson correlation. Moderated regression [24, 26–28] was subsequently used to evaluate the interaction between baseline speed (each UWS and MWS) and propulsion. The moderated regression models included three terms: prop_baseline_, either UWS_baseline_ or MWS_baseline_—depending on whether ΔUWS or ΔMWS were to be predicted, and the interaction between these two predictors. A significant interaction effect was explored using ±1 standard deviation values for the moderator variables [27]. Centered variables were used in the models, all statistical assumptions were ensured, and alpha was set to 0.05. An *a priori* power analysis revealed that with alpha = 0.05 and power = 0.80, 25 participants would be sufficient to detect a ∆R^2^ increase of 0.26 in a moderated regression model with 3 total predictors testing 1 interaction.

To further facilitate interpretation of a significant interaction in the moderated regression models, subgroup analyses were conducted. More specifically, changes in walking speed (i.e., ΔUWS or ΔMWS) were evaluated for four subgroups, each with *N* = 5 participants stratified according to prop_baseline_ and walking speed. First, the 10 participants with the highest prop_baseline_ were included in a *High Propulsion* (HP) subgroup and the 10 participants with the lowest prop_baseline_ were included in a *Low Propulsion* (LP) subgroup. Each of these subgroups were further divided into the 5 slowest (i.e., LP–Slow and HP–Slow) and fastest (i.e., LP–Fast and HP–Fast) participants--using either participants' UWS or MW﻿S, which corre﻿sponded to the dependent variable of interest. It is important to note that moderated regression models are able to examine interactions comprised of continuous variables, whereas these subgroup analyses categorize participants, which reduces power. Thus, only summary statistics are presented for these subgroups, with the average change observed in each subgroup compared to the established minimal clinically important difference (MCID) score for walking speed of 0.16 m/s [29].

## Results

Complete data were available for 25 of the 27 participants (Table 1). Propulsion data for two subjects were not available due to technical issues during collection. The average UWS_baseline_ was 0.65 ± 0.06 m/s, MWS_baseline_ was 0.84 ± 0.08 m/s, and prop_baseline_ was 5.40 ± 0.98 %bw. Table 2 details the characteristics of the subgroups studied.

**Table 1:** Participant characteristics

Participant Number	Sex	Age (y)	Time Since Stroke (y)	Side of Paresis	prop_baseline_	UWS_baseline_	MWS_baseline_	ΔUWS	ΔMWS
1	M	67	1.83	Left					
2	M	51	9.25	Left	6.07 %	0.86	1.08	0.24	0.32
3	M	58	9.17	Right					
4	M	63	7.99	Right	12.48 %	0.92	1.43	0.12	0.14
5	F	63	3.02	Right	10.99 %	0.94	1.09	0.05	0.50
6	F	65	22.90	Left	0.00 %	0.20	0.21	0.12	0.13
7	F	65	24.66	Left	4.78 %	0.70	1.06	0.03	0.03
8	M	71	5.83	Right	3.58 %	0.47	0.61	0.20	0.29
9	M	60	2.68	Left	3.69 %	0.41	0.40	0.24	0.34
10	M	66	1.58	Right	1.84 %	0.27	0.32	0.11	0.10
11	M	70	1.75	Left	2.61 %	0.47	0.50	0.11	0.20
12	F	65	1.25	Right	3.99 %	0.68	0.87	0.18	0.23
13	F	65	1.50	Right	1.13 %	0.51	0.82	0.15	0.24
14	F	54	4.58	Right	4.06 %	0.48	0.70	0.27	0.26
15	F	58	1.00	Right	0.05 %	0.29	0.36	0.17	0.18
16	M	46	0.67	Right	1.24 %	0.44	0.48	0.03	0.02
17	F	70	0.75	Left	4.31 %	0.34	0.36	0.16	0.16
18	M	69	2.86	Left	3.75 %	0.79	1.12	0.16	0.39
19	M	43	0.57	Left	7.26 %	0.61	0.85	0.33	0.23
20	M	58	0.59	Left	5.03 %	0.61	1.10	0.49	−0.07
21	M	68	0.77	Left	6.18 %	0.65	0.85	0.33	0.32
22	M	71	1.71	Left	19.91 %	1.16	1.12	0.28	0.59
23	M	55	1.66	Left	4.09 %	0.74	0.87	0.07	0.21
24	M	69	8.29	Right	3.40 %	0.72	0.86	−0.03	0.09
25	M	56	0.73	Left	0.53 %	0.33	0.40	0.17	0.21
26	F	56	3.51	Left	7.90 %	1.18	1.60	−0.09	0.05
27	M	25	1.70	Left	16.13 %	1.51	1.74	−0.44	−0.07

*Abbreviations*: *prop*_*baseline*_ peak propulsive force generated by the paretic limb at baseline, *UWS* usual walking speed, *MWS* maximum walking speed, *UWS*_*baseline*_ UWS at baseline, *MWS*_*baseline*_ MWS at baseline

**Table 2:** Subgroup characteristics

Outcome	Propulsion-Speed Subgroup			
	HP—Slow	HP—Fast	LP—Slow	LP—Fast
UWS (m/s)	0.69 ± 0.05	1.14 ± 0.11	0.30 ± 0.03	0.52 ± 0.05
MWS (m/s)	0.99 ± 0.06	1.40 ± 0.13	0.34 ± 0.04	0.65 ± 0.08
prop_baseline_ (%bw)	5.86 ± 0.45	13.5 ± 2.08	1.22 ± 0.70	1.16 ± 0.52
ΔUWS (m/s)	0.28 ± 0.08	−0.02 ± 0.12	0.16 ± 0.02	0.09 ± 0.04
ΔMWS (m/s)	0.28 ± 0.08	0.13 ± 0.12	0.19 ± 0.04	0.17 ± 0.05

*Abbreviations*: *HP*-*slow* high propulsion and slow walking speed subgroup, *HP*-*fast* high propulsion and fast walking speed subgroup, *LP*-*slow* low propulsion and slow walking speed subgroup, *LP*-*fast* low propulsion and fast walking speed subgroup, *UWS* usual walking speed, *MWS* maximum walking speed, *prop*_*baseline*_peak propulsive force generated by the paretic limb at baseline

### Changes in usual walking speed

**Fig. 1:**
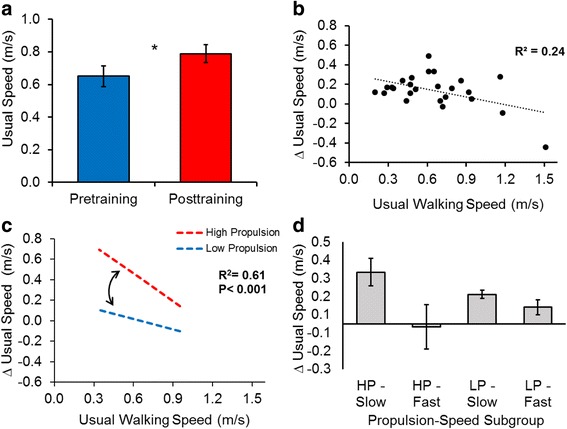
**a** Changes in usual walking speed (UWS) observed following 12 weeks of FastFES locomotor training (* *P* < 0.05). **b** Relationship between baseline UWS (x-axis) and ΔUWS (y-axis). **c** Interaction between baseline UWS and baseline paretic propulsion when predicting ΔUWS. The simple slopes presented were calculated using unstandardized regression coefficients (see Table 3), with moderation by baseline propulsion probed using 10.30 %bw (High Propulsion) and 0.50 %bw (Low Propulsion), which were, respectively, one standard deviation above and below the average for baseline propulsion. Although evaluated using these two values, it should be noted that baseline propulsion is treated as a continuous variable in the moderated regression model (represented by the curved arrow between regression slopes). **d** ΔUWS for different propulsion-speed subgroups. *Abbreviations: *HP-slow: high propulsion and slow walking speed subgroup; HP-fast: high propulsion and fast walking speed subgroup; LP-slow: low propulsion and slow walking speed subgroup; LP-fast: low propulsion and fast walking speed subgroup

Across participants, FastFES training produced an average ΔUWS of 0.14 ± 0.03 m/s (*P* = 0.001) (Fig. 1a) that remained at follow-up (*P* = 0.005). Participants’ UWS_baseline_ was negatively related with ΔUWS (*r* = -0.49, *P* = 0.01), explaining 24 % of the variance in ΔUWS (*R*^*2*^ = 0.24, *F*(*1*,*23*) = 7.06, *P* = 0.01) (Fig. 1b). The addition of prop_baseline_ to the model explained an additional 12 % of the variance in ΔUWS (∆*R*^*2*^ = 0.12, ∆*F*(*1*,*22*) = 4.00, *P* = 0.06). The addition of the UWS_baseline_ × prop_baseline_ interaction explained an additional 26 % of the variance in ΔUWS (∆*R*^*2*^ = 0.26, ∆*F*(*1*,*21*) = 14.25, *P* = 0.001). The final model (Table 3) explained 61 % of the variance in ΔUWS (*R*^*2*^ = 0.61, *F*(*3*,*21*) = 11.15, *P* < 0.001) and revealed that slower walkers who had higher baseline paretic propulsion achieved the largest ΔUWS (Fig. 1c). Subgroup analyses confirmed that the HP-Slow subgroup achieved the largest ΔUWS (0.28 ± 0.07 m/s)—a recovery of walking speed 1.75 times larger than the MCID of 0.16 m/s [29] (Fig. 1d). Whereas the LP-Slow subgroup achieved a ΔUWS that matched the MCID (0.16 ± 0.02 m/s), ΔUWS were not larger than the MCID in either the HP-Fast or LP-Fast subgroups.

**Table 3:** Moderated regression models predicting changes in walking speed

Models		Predictor Statistics			
Outcome	Statistics	Predictors	*b*	B	*p*
∆UWS	*R* ^*2*^ = 0.61 *F*(*3*, *21*) = 11.15 *P* = 0.000	Constant	0.21		0.000
		prop_baseline_ UWS_baseline_ UWS_baseline_ × prop_baseline_	−0.60 4.27 −5.48	−1.09 1.21 −0.72	0.000 0.001 0.001
∆MWS	*R* ^*2*^ = 0.49 *F*(*3*,*21*) = 6.69 *P* = 0.002	Constant	0.26		0.000
		prop_baseline_ MWS_baseline_ MWS_baseline_ × prop_baseline_	−0.27 3.59 −4.44	−0.69 1.11 −0.61	0.007 0.000 0.004

Regression models predicting posttraining changes in usual (UWS) and maximum (MWS) walking speeds

### Changes in maximum walking speed

**Fig. 2:**
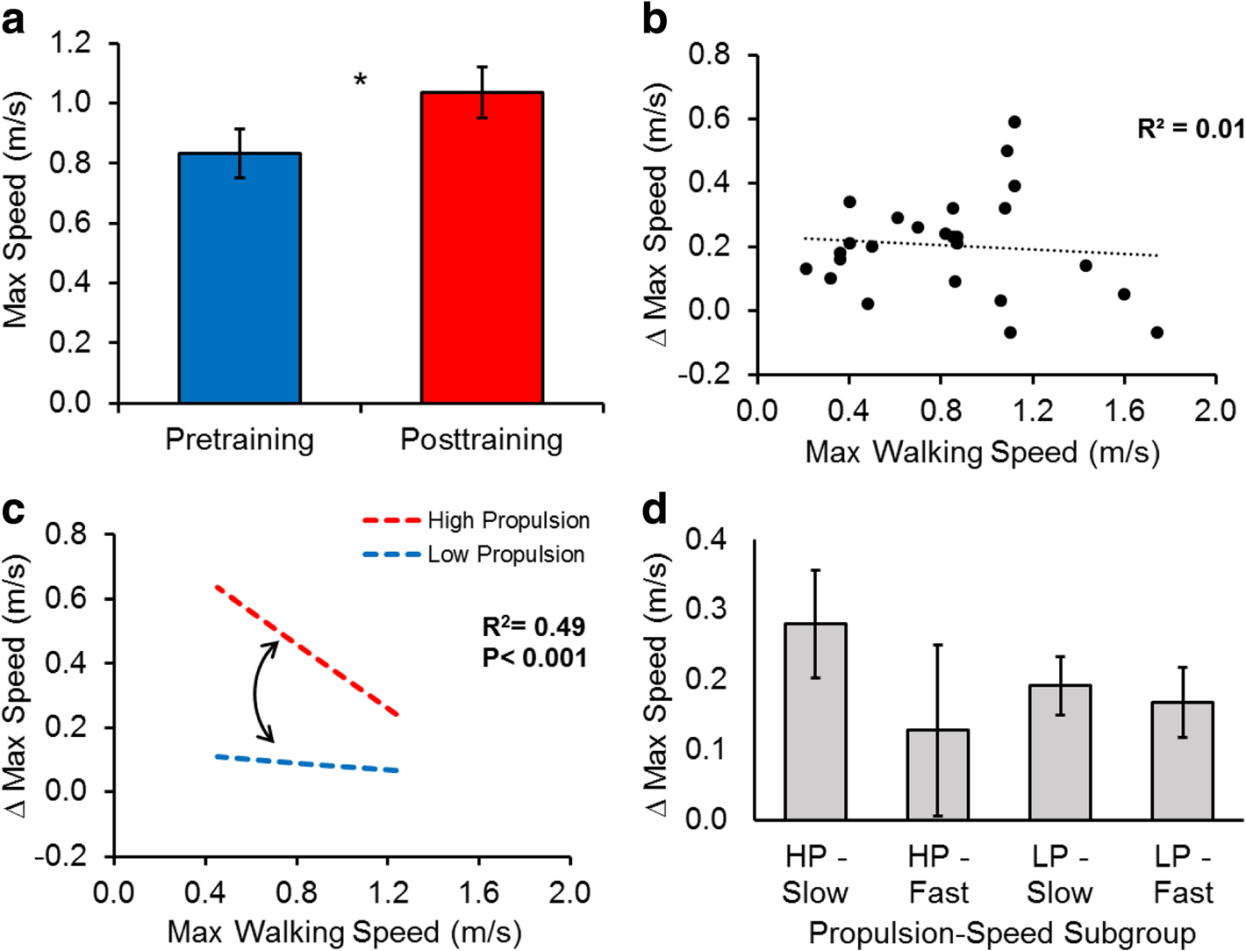
**a** Changes in maximum walking speed (MWS) observed following 12 weeks of FastFES locomotor training (* P <﻿ 0.0﻿5). **b** Relationship between baseline MWS (x-axis) and ΔMWS (y-axis). **c** Interaction between baseline MWS and baseline paretic propulsion when predicting ΔMWS. The simple slopes presented were calculated using unstandardized regression coefficients (see Table 3), with moderation by baseline propulsion probed using 10.30 %bw (High Propulsion) and 0.50 %bw (Low Propulsion), which were, respectively, one standard deviation above and below the average for baseline propulsion. Although evaluated using these two values, it should be noted that baseline propulsion is treated as a continuous variable in the moderated regression model (represented by the curved arrow between regression slopes). **d** ΔMWS for different propulsion-speed subgroups. *Abbreviations:* HP-slow: high propulsion and slow walking speed subgroup; HP-fast: high propulsion and fast walking speed subgroup; LP-slow: low propulsion and slow walking speed subgroup; LP-fast: low propulsion and fast walking speed subgroup

FastFES training produced an average ΔMWS of 0.20 ± 0.03 m/s (*P* < 0.001) (Fig. 2a) that remained at follow-up (*P* = 0.002). Participants’ MWS_baseline_ did not correlate with ΔMWS (*r* = -0.09, *P* = 0.670) (Fig. 2b). The addition of prop_baseline_ to the model explained an additional 23 % of the variance in ΔMWS (∆*R*^*2*^ = 0.23, ∆*F*(*1*,*22*) = 6.61, *P* = 0.017). The addition of the MWS_baseline_ × prop_baseline_ interaction explained an additional 25 % of the variance in ΔMWS (∆*R*^*2*^ = 0.25, ∆*F*(*1*,*21*) = 10.32, *P* = 0.004). The final model (Table 3) explained 49 % of the variance in ΔMWS (*R*^*2*^ = 0.49, *F*(*3*,*21*) = 6.69, *P* = 0.002), and, like the ΔUWS model, revealed that slower walkers with higher baseline paretic propulsion achieved the largest ΔMWS (Fig. 2c). Similar to ΔUWS, subgroup analyses confirmed that the HP-Slow subgroup achieved the largest ΔMWS (0.28 ± 0.08 m/s), followed by the LP-Slow subgroup (0.19 ± 0.04 m/s)—both gains that surpassed the MCID. In contrast to the ΔUWS results, the LP-Fast subgroup also achieved ΔMWS that surpassed the MCID (0.17 ± 0.05 m/s) (Fig. 2d). As with the ΔUWS results, ΔMWS were not larger than the MCID for the HP-Fast subgroup.

## Discussion

This report reveals that using only participants’ baseline walking speed to identify the best candidates for a targeted gait rehabilitation program is limited. A substantial increase in predictive power was observed in our regression models when including both participants’ baseline walking speed and paretic limb propulsive output—the primary target of the intervention studied. Indeed, the variance in functional recovery accounted for by the models increased dramatically for both speed outcomes when examining the interaction between speed and propulsion versus speed alone (i.e., from 24 to 61 % for ∆UWS and from 1 to 49 % for ∆MWS). Although walking speed is a robust and clinically meaningful metric that is commonly used to stratify participants in intervention studies [5, 7–10], the heterogeneity of motor impairment underlying similar poststroke walking speeds appears to reduce the utility of using walking speed to identify the appropriate candidates for a targeted gait intervention [16, 28]. These findings build on recent work calling for a quantification of the biomechanical deficits underlying walking function to guide clinical intervention [20, 30, 31] and support future work evaluating how different participant characteristics interact to influence the effects of poststroke locomotor training. This line of research is particularly critical for advancing the individualization of neurorehabilitation efforts in biomechanically-diverse populations.

Our finding of an interaction between participants’ baseline walking speed and paretic propulsion when predicting the effects of the FastFES intervention extends our research group’s efforts developing and testing this promising rehabilitation approach by identifying for whom the FastFES program is most effective. Ultimately, we found that the effects of FastFES were enhanced in slower walkers, especially those with higher baseline propulsion. Although the clinical utility of this finding may be limited due to the inability to directly measure gait kinetics in the clinic, several kinematic variables that can be measured in most clinical settings have been identified as being strongly linked to paretic propulsion (e.g., paretic trailing limb angle [32–34] and paretic and nonparetic step lengths [16, 35]). Moreover, there is growing interest in developing low cost, wearable technology solutions to enable measurement of metrics such as propulsion outside of the laboratory [30, 31]. Further research and development in this area appear worthwhile.

It is interesting to note that FastFES training was most effective in those with larger baseline paretic propulsion. As an intervention specifically designed to improve paretic propulsion, it would have been conceivable to hypothesize that FastFES would be most effective in those with lower levels of baseline propulsion. Nonetheless, it is important to note that, in this study, even those with the largest baseline propulsion were still markedly impaired in their ability to generate propulsion via the paretic limb. Indeed, only one of the participants studied presented with baseline paretic propulsion comparable to the average observed in neurologically-intact, elderly subjects (20 %BW, see Table 1) [36]. One explanation for why FastFES training was not as effective in those with low levels of baseline propulsion is that FastFES training may not be sufficient to overcome particular pre-existing compensatory walking strategies that may be characterized by very low paretic propulsion during walking. That is, for participants largely dependent on compensatory strategies known to impair the propulsive-force generating ability of the paretic limb [34, 37–39], FastFES training may not provide a sufficient stimulus to alter this walking strategy. Ultimately, our finding that FastFES training was more effective in participants with small-to-moderate levels of paretic propulsion may suggest that FastFES training is able to enhance an already present, but impaired, propulsion-based walking strategy, but may not be as appropriate for participants with low baseline propulsion due to their reliance on propulsion-impairing compensatory strategies. Indeed, recent work has demonstrated that in untrained individuals poststroke, the better their ability to generate more paretic propulsion when made to walk faster, the larger the gains in paretic propulsion following targeted gait training [40]. An alternative explanation is that participants with very low baseline levels of paretic propulsion may not have the capacity to walk via propulsion due to insufficient neural substrate. For these individuals, any training centered on improving paretic propulsion (e.g., FastFES) may not be appropriate. Recent work showing that reduced corticomotor input to the paretic plantarflexors is related to asymmetrical interlimb propulsive strategies during walking [41] supports this alternative hypothesis. Future investigation into how the ability to activate the paretic plantarflexors influences the effects that targeted locomotor training has on the recovery of more physiological gait mechanics and walking function is warranted.

## Conclusions

This report demonstrates the value of investigating how the baseline characteristics of individuals poststroke interact to influence the effects of targeted locomotor training. For a population as heterogeneous as those in the chronic phase of stroke recovery, a better understanding of such interactions is critical for advancing personalized neurorehabilitation. The findings of this study suggest that the factors able to predict the effects of an intervention may be suitably defined by the targets of the intervention. Indeed, for FastFES locomotor training, knowledge of how baseline paretic propulsion interacted with baseline walking speed substantially improved the ability to predict the recovery of walking speed following intervention.

## Abbreviations

FastFES: A 12-week poststroke locomotor rehabilitation program combining fast walking with functional electrical stimulation to the paretic ankle muscles during walking
HP-fast: High propulsion and fast speed subgroup
HP-slow: High propulsion and slow speed subgroup
LP-fast: Low propulsion and fast speed subgroup
LP-slow: Low propulsion and slow speed subgroup
MWS: Maximum walking speed
MWS_baseline_: MWS at baseline
paretic propulsion: Peak anterior ground reaction force generated by the paretic limb during walking
prop_baseline_: Paretic propulsion at baseline
UWS: Usual walking speed
UWS_baseline_: UWS at baseline
